# Impact of Fe^2+^ and Shear Stress on the Development and Mesoscopic Structure of Biofilms—A *Bacillus subtilis* Case Study

**DOI:** 10.3390/microorganisms10112234

**Published:** 2022-11-11

**Authors:** Luisa Gierl, Harald Horn, Michael Wagner

**Affiliations:** 1 Water Chemistry and Water Technology, Engler-Bunte-Institut, Karlsruhe Institute of Technology, Engler-Bunte-Ring 9a, 76131 Karlsruhe, Germany; 2 German Technical and Scientific Association for Gas and Water (DVGW) Research Site at Karlsruhe Institute of Technology, Water Chemistry and Water Technology, Engler-Bunte-Ring 9a, 76131 Karlsruhe, Germany; 3 Institute of Biological Interfaces (IBG-1), Karlsruhe Institute of Technology, Hermann-von-Helmholtz-Platz 1, 76344 Eggenstein-Leopoldshafen, Germany

**Keywords:** *Bacillus subtilis* biofilms, biofilm structure, biofilm development, biofilm stability, optical coherence tomography, iron

## Abstract

Bivalent cations are known to affect the structural and mechanical properties of biofilms. In order to reveal the impact of Fe^2+^ ions within the cultivation medium on biofilm development, structure and stability, *Bacillus subtilis* biofilms were cultivated in mini-fluidic flow cells. Two different Fe^2+^ inflow concentrations (0.25 and 2.5 mg/L, respectively) and wall shear stress levels (0.05 and 0.27 Pa, respectively) were tested. Mesoscopic biofilm structure was determined daily in situ and non-invasively by means of optical coherence tomography. A set of ten structural parameters was used to quantify biofilm structure, its development and change. The study focused on characterizing biofilm structure and development at the mesoscale (mm-range). Therefore, biofilm replicates (*n* = 10) were cultivated and analyzed. Three hypotheses were defined in order to estimate the effect of Fe^2+^ inflow concentration and/or wall shear stress on biofilm development and structure, respectively. It was not the intention to investigate and describe the underlying mechanisms of iron incorporation as this would require a different set of tools applied at microscopic levels as well as the use of, i.e., omic approaches. Fe^2+^ addition influenced biofilm development (e.g., biofilm accumulation) and structure markedly. Experiments revealed the accumulation of FeO(OH) within the biofilm matrix and a positive correlation of Fe^2+^ inflow concentration and biofilm accumulation. In more detail, independent of the wall shear stress applied during cultivation, biofilms grew approximately four times thicker at 2.5 mg Fe^2+^/L (44.8 µmol/L; high inflow concentration) compared to the low Fe^2+^ inflow concentration of 0.25 mg Fe^2+^/L (4.48 µmol/L). This finding was statistically verified (Scheirer–Ray–Hare test, ANOVA) and hints at a higher stability of *Bacillus subtilis* biofilms (e.g., elevated cohesive and adhesive strength) when grown at elevated Fe^2+^ inflow concentrations.

## 1. Introduction

Biofilms are aggregates composed of microorganisms, extracellular polymeric substances (EPS) as well as extracellular DNA. They appear at interfaces in various watery/moist environments [1]. Within the last decade, more attention has been paid to biofilms due to their unique properties and potential applications as productive systems [2,3]. On the one hand, these communities have several beneficial features, e.g., (i) cleaning wastewater [4]; (ii) producing valuable (platform) chemicals [5], (iii) methane (as fuel) [6] or (iv) bioplastics [7], too. On the contrary, biofilms can have adverse effects, i.e., by blocking industrial settings such as water pipes and membranes [8,9] and inducing bio-corrosion [10]. Moreover, biofilms in medical settings cultivate organs as well as implants causing for example an elevated (antimicrobial) drug resistance and (chronic) infections, respectively. More detailed information is provided elsewhere [11,12].

Understanding biofilm proliferation, development, processes and behavior under certain conditions is crucial in order to optimize and control the cultivated technological or biological system. Direct analysis of biofilm behavior or rather structure can be performed using different imaging techniques such as confocal laser scanning microscopy (CLSM), scanning electron microscopy (SEM) or atomic force microscopy (AFM) [8,13,14,15]. While CLSM can be used for investigating the biofilm matrix composition (e.g., DNA and EPS) in a range of several micrometers, SEM together with energy-dispersive X-ray spectroscopy (EDX) examines biofilms up to 1 nm resolution including their elemental composition. Atomic force microscopy (AFM) determines for instance the adhesion forces between the biofilm and the substratum as well as the cohesive strength at single cell level and below [8,13]. Optical coherence tomography (OCT) is an application that is becoming increasingly relevant for the analysis of biofilms’ mesoscopic structure as seen from the increasing number of publications [16,17,18,19,20,21]. Advantages of OCT among other imaging techniques are the high optical resolution together with a fast acquisition of 3D datasets of translucent tissues and materials in situ despite large representative volumes [22]. The mesoscale of biofilms is valuable for, e.g., modeling permeate fluxes in membrane systems [9,23] or modelling substrate turnover in biofilm reactors [20,24].

Specific research questions require the application of adequate imaging modalities. Microscopic techniques such as AFM, SEM and CLSM provide detailed information about biofilm composition, rheological properties and microscopic structure (e.g., cell distribution). However, analyzing sufficiently large biofilm structures in order to derive representative results is time consuming and thus may alter the biofilm structure. Moreover, analysis of biofilm replicates (*n* > 3) is a requirement in order to draw valid conclusions about relations between cultivation conditions, structural features and underlying processes [25]. For those reasons, OCT has been selected as the visualization technique in combination with an extensive biofilm structure analysis.

Nutrients and hydrodynamics are some of the main effectors in the biofilm lifecycle. Several studies have been performed which focus on the influence of different ions and flow velocities on biofilm behavior [26,27,28,29,30,31,32,33]. Bivalent cations such as Ca^2+^ and Mg^2+^ are known to promote the growth and stability of biofilms [26,29,30,31]. Additionally, iron (Fe^2+^) may be of concern regarding biofilm development. Iron is an essential trace element and component of iron-sulfur complexes in various enzymes. Moreover, a couple of bacteria utilize Fe^3+^ as an electron acceptor within the respiratory chain [34]. Furthermore, iron is essential for almost all living organisms and forms a cofactor in many cellular proteins, which are involved in electron transport, detoxification of reactive oxygen species (ROS) or DNA synthesis [35]. For that reason, at least a minimum of iron availability should be necessary for the maturation process of biofilms. The role of iron has been studied by several research groups, who have mainly applied pathogenic *Pseudomonas aeruginosa* or static biofilms (e.g., agar plate and microtiterplate biofilms). Available results did not approve an effect of iron ions in the cultivation medium on biofilm growth and structure [36,37,38,39,40,41]. Additionally, most studies have merely focused on the performance of those biofilms instead of physical structure - although structure and function are closely linked to each other. For instance, Möhle et al. described a positive effect on the stability of biofilms grown in a rotating disc reactor when higher amounts of iron sulfate (10 mg/L) were available [42]. Further studies showed that a limitation of iron [43,44] as well as an excess of iron [39,41,45] in the growth environment inhibited the formation and development of biofilms in contrast to suspended cells [46].

In this study, the effect of Fe^2+^ at two levels (0.25 and 2.5 mg/L Fe^2+^, respectively; 4.48 µmol/L and 44.8 µmol/L Fe^2+^, respectively) in the cultivation medium on the structure, development and maturation of *Bacillus subtilis* biofilms cultivated in mini-fluidic flow cells was investigated. Additionally, the effect of two wall shear stress conditions (0.05 and 0.27 Pa, respectively) on the biofilm structure was evaluated since it has often been reported [28,47] that a higher shear stress causes more compact and flat biofilms whereas a low shear stress results in fluffy, patchy biofilms.

By testing both parameters, this work strengthens the fundamental knowledge about biofilm physical structure and driving factors. Furthermore, it highlights Fe^2+^ as a supplement controlling biofilm structure and development, maturation at the mesoscale as well as cohesive and adhesive properties. It is important to know at this point that the study was not conducted in order to provide a mechanistic understanding of iron incorporation into the biofilm matrix.

## 2. Materials and Methods

### 2.1. Biofilm Cultivation

Biofilms were cultivated in custom-made flow cells composed of sticky-Slides (sticky-Slide I 0.4 Luer, ibidi GmbH, Martinsried, Germany; https://ibidi.com/img/cms/products/labware/sticky_slides/S_8XXXX_sticky_I_Luer/IN_8XXXX_Sticky_Luer.pdf (accessed on 3 November 2022) glued to PVC slides serving as substrata. Sticky-Slides are made from transparent plastic and serve as the cover of the flow cell forming a flow channel with the size of 50 × 5 × 0.45 mm^3^ (length × width × height, thickness of the sticky-Slide = 1 mm). A number of N = 10 flow cells were operated for each condition in parallel at volumetric flow rates of Q = 1 mL/min and Q = 5 mL/min, respectively (equal means flow velocities and wall shear stress levels of u = 0.75 cm/s and τ_wall_ = 0.05 Pa as well as u = 3.75 cm/s and τ_wall_ = 0.27 Pa, respectively). These hydrodynamic conditions were chosen according to Paul et al., (2012) who proposed a range of τ_wall_ = 0.1–13 Pa as high shear stress and a shear stress of 0.01 Pa as a very low one. Thus, 0.05 Pa refers to low shear stress conditions whereas the approx. 5-fold of 0.27 Pa refers to a high shear stress level.

Flow cells were inoculated with *Bacillus subtilis* (NCBI 3610) pre-cultures grown at 37 °C overnight in Erlenmeyer flasks in Lysogeny Broth (LB-Miller, 10 g/L NaCl) medium. Before inoculation, *Bacillus subtilis* pre-cultures were grown to the exponential phase. After reaching exponential growth, 10 mL of the pre-culture was mixed with a minimal salt glycerol medium in a mixing ratio of 1:500. This cell suspension was used to inoculate the flow cells.

The cultivation medium was adapted from (Wang, Wang, and Hao, 2015) and contained (concentration in mg/L): MnCl_2_ • 4 H_2_O (10), L-phenylalanine (5), glycerol (5), MgCl_2_ • 6 H_2_O (4) and L-tryptophane (3.5) with diverging concentrations of FeCl_2_ • 4 H_2_O (≙ 0.25 and 2.5 mg/L Fe^2+^; c(Fe^2+^) used by Wang, Wang and Hao (2015) was 1.23 mg/L Fe^2+^) in tap water. Salt concentrations of the tap water of Karlsruhe are accessible from the homepage of the Stadtwerke Karlsruhe (https://www.stadtwerke-karlsruhe.de (accessed on 28 December 2020) and contained (mg/L): Ca (112), Na (11), Mg (9.7), Si (5.4), K (1.7), P (<0.01), Fe (<0.01) and Mn (<0.005). The iron(II) concentration of 0.25 mg/L Fe^2+^ was chosen to be below the elsewhere used concentration of 1.23 mg/L Fe^2+^ [48]. It was assumed that a low inflow concentration of Fe^2+^ would cause slow growing/accumulating biofilms. In contrast, the 10-fold inflow concentration of 2.5 mg/L Fe^2+^ was chosen in order to enhance biofilm development (e.g., growth/accumulation).

Flow cells were flushed with the inoculum for 15 min. Afterwards, flow was stopped for 1 h allowing bacteria to settle. Then biofilm cultivation was conducted in flow-through mode at an ambient temperature (21–22 °C) and a neutral pH-value of 7. Biofilm development 25 mm downstream of the inlet was monitored daily for ten consecutive days by means of OCT using the EvoBot platform [20,49]. Briefly, the EvoBot platform allows the automated positioning of the OCT scanning probe as well as the acquisition of three-dimensional OCT datasets similar to the platform described by Depetris et al. [50]. Thereby, operator-related errors during dataset acquisition are reduced.

An overview of the conducted experiments is provided in Table 1 whereas the utilized flow cell setup is explained in detail in Gierl et al. [25].

### 2.2. Chemical Characterization of Biofilm by Means of ATR-IR and ICP-OES

Attenuated total reflectance infrared spectroscopy (ATR-IR) was utilized for analyzing present iron derivatives within the biofilm. Before using the spectrometer with diamond crystal (Vertex 70, Bruker Corporation, Billerica, MA, USA), the ATR unit was flushed with nitrogen for approx. 30 min. Each measurement included a performance and operational qualification test. Dried biofilm samples (24 h, 105 °C) were placed on the ATR crystal. Spectra were acquired between 4000 and 200 cm^−1^ with an averaging of 30 scans. Collected data were evaluated in the spectroscopy software OPUS (Version 7.5, Bruker Corporation, Billerica, MA, USA). Identification of spectra (e.g., determination of FeOOH modification) was by comparison with the literature and against reference databases (confer Figure S2, SI for band assignment).

The iron accumulated inside the biofilm was quantified by means of inductively coupled plasma optical emission spectroscopy (ICP-OES; ICP-OES 5110, Agilent Technologies Incorporation, Santa Clara, CA, USA) after microwave extraction (Mars 5, CEM GmbH, Kamp-Lintfort, Germany). Additionally, biofilm wet and dry mass was determined gravimetrically.

### 2.3. Optical Coherence Tomography and Image Processing

A spectral domain optical coherence tomograph (GANYMEDE system, Thorlabs GmbH, Dachau, Germany) with an optical resolution of 8 × 8 × 2.1 µm^3^ (x × y × z, LSM03 objective lens) in water (n = 1.33) was used to monitor biofilm development. OCT 3D datasets (A-scan averaging = 3) with a size of 7 × 6 × 0.5 mm^3^ were acquired on a daily basis. Image post-processing included the calculation of structural biofilm parameters. Prior to dataset analysis, OCT datasets were cropped to a volume of 7 × 5 × 0.25 mm^3^ in order to exclude autocorrelation artifacts. Thus, only half of the flow channel height (the first 0.25 mm above the substratum) was analyzed.

A mean filter with a radius of 2 px was applied and binary datasets were generated using Fiji [51]. Substratum coverage (SC), mean biofilm thickness (${\bar{L}}_{F}$), textural entropy (TE), kurtosis $\left(R_{KU}\right)$, skewness ($R_{SK}$), fractal dimension (FD), angular second moment (ASM), inverse difference moment (IDM) as well as average horizontal (AHRL) and average vertical run lengths (AVRL) were calculated from binary datasets according to [16,20,52] and by the use of the MiToBo plugin (Fiji) for biofilms [53]. In-house macros were applied to render topographic representations of OCT volume scans (e.g., height maps representing the bulk-biofilm interface; see [20,52]).

An overview of all structural parameters and their calculation is given in [20,41,54]. Parameters SC, $R_{KU}$ and $R_{SK}$ were analyzed using Fiji’s plugin function “Analyze”. An overview regarding the interpretation of the structural biofilm parameters is presented below (see Table 2).

### 2.4. Analysis of Variance (ANOVA) and Scheirer–Ray–Hare

Grubb’s tests and normality tests (Shapiro–Wilk) were performed in Origin to identify and discard outliers (OriginPro, Version 2018G, OriginLab 275 Corporation, Northhampton, MA, USA). Two factorial variance analyses with measuring repetitions were performed to evaluate the influence of Fe^2+^ and (wall) shear stress on biofilm development and structure. In case of non-normality, a Scheirer–Ray–Hare test was applied. Three hypotheses were picked with the following predications:

*H_1_: No differences in structure due to usage of different flow velocities* u

*H_2_: No differences in structure due to usage of different iron concentrations* c

*H_3_: No correlation between both parameters* u *and* c

To determine the approval or rejection of the hypotheses, calculated p-values were compared to a significance level of α < 0.01, whereby values p < α describe the rejection of the individual hypothesis. Variance analyses were performed in Excel (Excel version 15.11, Microsoft Corporation, Redmond, DC, USA).

## 3. Results and Discussion

### 3.1. Structural Differences in OCT-Imaged Biofilms

**Figure 1:**
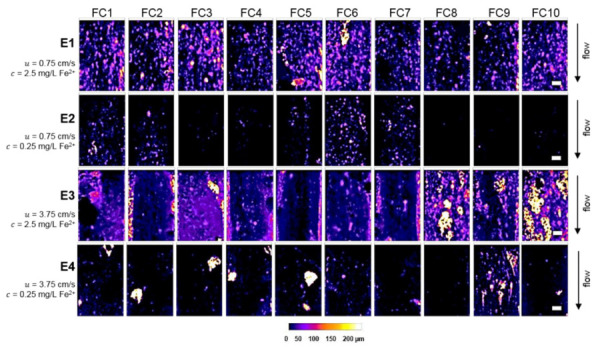
Biofilm height maps of day 10 comparing biofilms of each condition. The calibration bar resembles the biofilm height ($L_{F}$) in µm. The scale bar equals 1 mm. Experiments E1–E4 are explained in Table 1. Abbreviation FC refers to flow cell.

Four growth conditions were applied to biofilms that differed in wall shear stress (e.g., different constant volumetric flow rates Q) and the Fe^2+^ concentrations c; see Table 1. Again, the aim of the study was the identification of a dependency of biofilm development and structure in terms of inflowing Fe^2+^ as well as of different shear stress conditions. Figure 1 illustrates height maps showing the topography of the developed biofilms at day 10 for each flow cell under each applied condition. Topographic representations of all flow cells on each day are given in Supplementary Information Figure S1.

Figure 1 reveals biofilm growth in all flow cells (FC). It is worth noting that FC 9 of E4 had a different appearance among the other flow cells for these experimental conditions. Visible similarities were observed for E3 in flow cells FC 5 to FC 8 and FC 10, respectively. Experiment E1 (c = 2.5 mg/L Fe^2+^, u = 0.75 cm/s) led to biofilms with a mean biofilm thickness ${\bar{L}}_{F}$ = 75 µm in all replicates. Additionally, under condition E1, biofilm aggregates of $L_{F}$ up to 200 µm were established and the substratum was covered with heterogeneous aggregates, ranging from smaller and spherical colonies to longer (>2 mm) and elliptic colonies. In experiment E2 (c = 0.25 mg/L Fe^2+^, u = 0.75 cm/s) less biofilm developed: only flow cells FC 1, FC 2 and FC 5 to 7 were covered with biofilm (${\bar{L}}_{F}$ = 50 µm). In comparison to E1, biofilm growth within E2 was delayed and only small colonies of minor widths and lengths, as well as of minor heights, accumulated. In E3 (c = 2.5 mg/L Fe^2+^, u = 3.75 cm/s) in turn, the biofilm appeared dense at day 10 of the cultivation. Similar to E2, bacterial colonization was first visible around days 3 and 4. Here, accretion within the flow chamber took place from the walls to the center of the FC. In E4 (c = 0.25 mg/L Fe^2+^, u = 3.75 cm/s), coverage of the substratum was again low as in E2 and only a few colonies randomly developed with high biofilm thicknesses of ${\bar{L}}_{F}$ = 200 µm.

**Figure 2:**
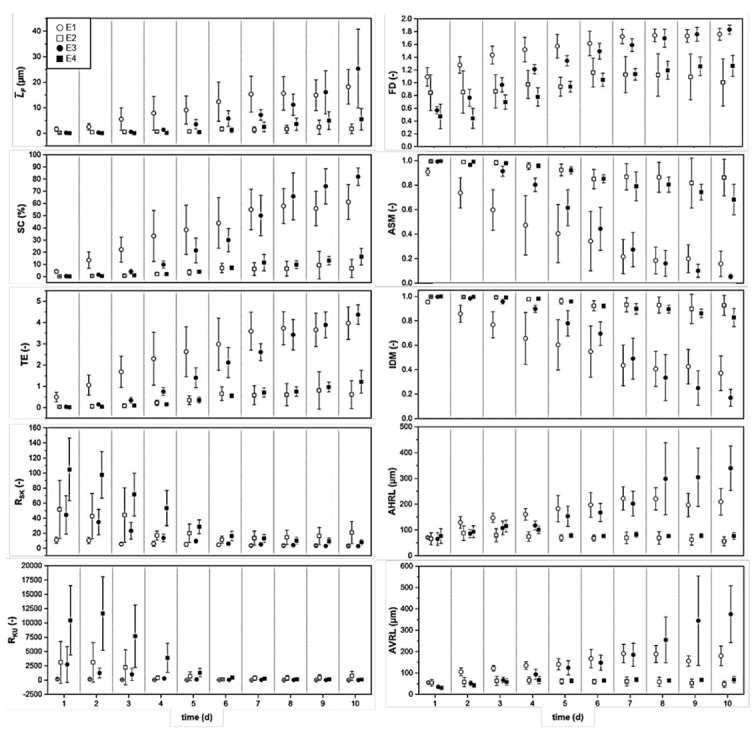
Comparison of the structural biofilm parameters substratum coverage (SC), mean biofilm thickness (${\bar{L}}_{F}$), textural entropy (TE), kurtosis $\left(R_{KU}\right)$, skewness ($R_{SK}$), fractal dimension (FD), angular second moment (ASM), inverse difference moment (IDM) as well as average horizontal (AHRL) and average vertical run lengths (AVRL) over the course of the experiments (10 days). Inoculation took place on day 0. An overview and meaning of the parameters are provided in Table 2. *n* = 10 replicates; E1/E3, ◯● ≙ $c_{Fe}$_2+_ = 2.5 mg/L Fe^2+^; E2/E4, □⬛ ≙ $c_{Fe}$_2+_ = 0.25 mg/L Fe^2+^; opened data points ≙ *u* = 0.75 cm/s; closed data points ≙ *u* = 3.75 cm/s.

Figure 1 already provides visible differences in growth patterns for different experimental conditions. However, for the quantification of the influence of flow velocity (shear stress) and iron (Fe^2+^) dosage on biofilm structure and development, several structural parameters were calculated (confer Section 2). Those are presented in Figure 2 and discussed in the following.

Notice, parameters (e.g., ${\bar{L}}_{F}$) may appear different when comparing different experimental conditions such as iron(II) concentration in the inflow or wall shear stress. However, standard deviation (expressed by the symmetric error bars) of replicate experiments might be as high as the recognized difference between the plotted mean values. This has to be considered for the correct interpretation of presented results.

In Figure 2, the development of these 10 different structural biofilm parameters for all conditions E1–E4 is illustrated. Their meaning is explained in Table 2. As already visible in Figure 1, the effect of the high (E1 + E3, c = 2.5 mg/L Fe^2+^) and the low iron(II) concentrations (E2 + E4, c = 0.25 mg/L Fe^2+^) is visible. While mean biofilm thickness ${\bar{L}}_{F}$ and substratum coverage SC of E1 and E3 showed a steady increase until the end of the experiment, those parameters stayed at a minimum level in E2 and E4. A similar trend was distinct for the parameters including textural entropy TE, average horizontal AHRL and average vertical run length AVRL. Additionally, the stated parameters of E3 (c = 2.5 mg/L Fe^2+^, u = 3.75 cm/s) exceeded those of E1 (c = 2.5 mg/L Fe^2+^, u = 0.75 cm/s) at the end of the experiment (from day 8 to day 10). The same trend was shown for biofilms grown with c = 0.25 mg/L Fe^2+^, whereas the structural parameters in E4 (u = 3.75 cm/s) exceeded those of E2 (u = 0.75 cm/s). While mean biofilm thickness ${\bar{L}}_{F}$ and substratum coverage SC indicated an early accumulation of biofilms with high biofilm volume in E1 and E3 (c = 2.5 mg/L Fe^2+^), higher values of textural entropy TE exhibited more heterogeneous biofilms. Thus, biofilms grown with 2.5 mg/L Fe^2+^ showed more differentiated and partially distributed structures. Furthermore, average run lengths AHRL and AVRL of biofilms in E1 and E3 displayed longer and wider biofilm aggregates resulting in the largest aggregates regarding biofilm volume in E3 (c = 2.5 mg/L Fe^2+^, u = 3.75 cm/s) (compare Figure 1).

As seen from the structural parameters skewness $R_{SK}$ and kurtosis $R_{KU}$, biofilms in E4 (c = 0.25 mg/L Fe^2+^, u = 3.75 cm/s) displayed random distributed and high biofilm hills with a low substratum coverage SC at the beginning of the experiment. From day 5 to the end of the experiment (day 10), values of $R_{SK}$ and $R_{KU}$ under all conditions approximated and stayed near zero. Thereby, a homogeneous distribution of biofilm as well as an equalized ratio of colonies with either low or high biofilm thickness ${\bar{L}}_{F}$, except for E2 (c = 0.25 mg/L Fe^2+^, u = 0.75 cm/s) where only several high and small biofilm aggregates occurred, was confirmed (compare Figure 1). Again, structural biofilm parameters including fractal dimension FD, angular second moment ASM and inverse difference moment IDM demonstrated a lucid differentiation between biofilms grown with c = 0.25 mg/L Fe^2+^ and c = 2.5 mg/L Fe^2+^. Higher values of FD up to 1.8 in E1 and E3 (c = 2.5 mg/L Fe^2+^) explained the more irregular surfaces of the aggregates (compare Table 2). Here, the parameter ASM described a change in growth direction (additional growth in y-direction (width)) and IDM proved additional growth in width, since distances between cell clusters were minimized at the end of the experiment, compared to E2 and E4 (c = 0.25 mg/L Fe^2+^).

As can be seen, biofilm thickness ${\bar{L}}_{F}$ and/or surface coverage SC, which are typically used for characterization of biofilm structure, do not provide the complete information on biofilm development. Therefore, only the joint consideration of all evaluated structural parameters led to a conclusive overview of biofilm development over the cultivation period of 10 days. Figure 2 shows that at the beginning, biofilms cultivated at τ_wall_ = 0.27 Pa (u = 3.75 cm/s, E3 + E4) developed more slowly. Similar observations were made by Paul et al. who determined thinner biofilms at higher shear stress levels due to biofilm detachment through erosion [28]. However, towards the end of the experiment (days 8 to 9) those biofilms (E3 + E4) showed fewer fluctuating values with respect to ${\bar{L}}_{F}$, SC, ASM and IDM in relation to biofilms in E1 and E2 (u = 0.75 cm/s). With regard to the different iron(II) concentrations, more unambiguous differences in mostly all structural parameters could be identified (correlation of E1 + E3 and E2 + E4). These results confirm the positive influence of Fe^2+^ on biofilm accumulation (growth) and differentiation into secondary biofilm structures (also known as “fungi-like” structures stated in several publications [55,56,57,58]. Likewise, Körstgens et al. [59] showed, that bivalent cations (e.g., Ca^2+^) enhanced the stability of the biofilm matrix in *Pseudomonas aeruginosa* biofilms which could explain the increased adhesion and biofilm accumulation in E1 and E3 (c = 2.5 mg/L Fe^2+^) of the presented study. However, as EPS were not characterized chemically, a confirmation of alginate-like components within the EPS known to be stabilized by Ca^2+^ is lacking. In the study by Möhle et al. [42], increased concentrations of iron(II) sulfate in the nutrient medium (c = 10 mg/L equal to 3.6 mg Fe^2+^/L) prevented the sloughing of microbial biofilms (activated sludge) in a rotating disk reactor. Furthermore, the authors documented the dependence of the biofilm thickness from the substrate concentration c as well as from the shear stress on the biofilm surface. Beyond, in studies with iron-complexing agents, it was found that a minimal concentration of soluble iron is necessary for the formation of *P. aeruginosa* biofilms in flow cells [43,60,61]. Thereby, one theory is that iron regulates the surface motility of the bacteria and again promotes the biofilm formation by stabilizing the EPS matrix, which mainly consists of negatively charged polymers [37,43,44,45].

These studies further showed that an excess of iron concentrations inhibits biofilm formation, too, since the release of DNA from dead *P. aeruginosa* cells is suppressed. This release is an important structural component of biofilms [45,61]. However, an ideal iron concentration in the culture medium cannot easily be determined. While Berlutti et al. [37] define “high” iron concentrations in a range of 0.55–5.5 mg/L as positive in terms of aggregation and manipulation of biofilm development and structure in different reactor systems and tests, Yang et al. [61] reported an inhibition of biofilm growth in microtiter plates and flow cells in this concentration range. In the present study, the inhibitory effects of high iron concentrations on biofilm development could not be proven. Presumably, an addition of iron (Fe^2+^) does not stimulate every bacterial biofilm system, or possibly, optimum iron amounts can vary among different biofilm species. Nevertheless, Weinberg [44] confirmed that zinc, manganese and iron have key functions in the biochemical as well as in the morphological conversion of pro- and eucaryotes, respectively. Since soil carries high amounts of iron, a positive influence on growth of the used soil bacterium *Bacillus subtilis* could be demonstrated in the present study [62,63,64].

Likewise, the influence of hydrodynamics on biofilms is well-known and has been documented in several studies [21,27,28,31,32,33,65,66,67]. These studies verified the formation of streamers at higher flow velocities meaning increased growth in length, which is best visible in E3 for FC 8 and E4 for FC 9 (u = 3.75 cm/s, see Figure 1 and Figure 2 AHRL). Moreover, several studies contain statements about positive correlations between the viscoelastic properties and strength of the cell clusters and the hydrodynamics growth conditions [13,31,32,68,69,70,71].

### 3.2. ANOVA-Confirmed Effects of Iron(II) on Biofilm Structure

Two factor-based variance analyses were performed to verify the results of the structural biofilm parameters given in Figure 2. These evaluations determine to what extent a correlation of the conditions (c, u) took place. Furthermore, they provide insight on the differentiation of structural biofilm parameters between conditions with statistical certainty.

A p-value above the significance level $\alpha_{0.01}$ ked to an acceptance of the hypothesis and acknowledged that no differences in biofilm structure could be estimated due to the chosen condition. This was shown by both the ANOVA and the Scheirer–Ray–Hare test for all structural parameters in hypothesis H_1_ (Table 3).

With a minimum value of the skewness parameter with $R_{SK}$ (p = 0.07 > α) up to a maximum value of the substratum coverage SC (p = 0.84 > α), there was no difference between the low (u = 0.75 cm/s) and high flow velocity (u = 3.75 cm/s). On closer consideration of $R_{SK}$ in Figure 2, it is clear that this parameter showed the lowest p-value in H_1_ because there was no overlapping of the standard deviations of E1 + E3 as well as of E2 + E4 at the end of the experiment. This documents that the volumetric flow rate and resulting shear stress $\tau_{w}$ had a slight influence on the biofilms regarding skewness $R_{SK}$ and the fractal dimension FD. Nevertheless, this could not be confirmed with statistical significance compared to the influence of Fe^2+^ on the biofilm aggregates (see Table 3, H_2_). In comparison to Figure 2, Fe^2+^ had a positive effect on the mean biofilm thickness ${\bar{L}}_{F}$ and substratum coverage SC, which basically means on biofilm accumulation.

In contrast, diagrams of skewness $R_{SK}$ and kurtosis $R_{KU}$ revealed the similar values for conditions E1–E4 at the end of the experiments. This was confirmed by the ANOVA for $R_{SK}$ with the closest value to α (p = 0.18·10^−2^ < α) in H_2_. The value of $R_{KU}$, on the other hand, was far from the significance level (p = 0.56·10^−5^ < α), although the experimental course of $R_{KU}$ resembled the development of $R_{SK}$ in Figure 2. One reason for this might have been the use of the Scheirer–Ray–Hare test, which is generally considered to be less accurate than the ANOVA [72]. The results of H_3_ again showed, as was evident from H_1_, p-values above the significance level α. This supports the assumption that the influence of the wall shear stress and the addition of Fe^2+^ do not correlate with one another. Solely, the value of the average horizontal run length AHRL was relatively approximate to the level of significance (p = 0.04 < α). An explanation is given by the non-changing direction of water flow in the channel: preferably, the aggregates are growing in the x-direction if sufficient addition of Fe^2+^ is present, as the flow is unidirectional.

The investigation of the fluid–structure interaction demonstrates the need to analyze a large number of biofilm structural parameters since the evaluation of one parameter may (possibly) not be sufficient to determine differences but instead may determine dependencies and correlations (e.g., $R_{SK}$, $R_{KU}$). Additionally, the choice of a statistical test such as an ANOVA will prove the occurrence of underlying relations.

### 3.3. Influence of Fe^2+^ Inflow Concentration and Precipitated Fe Compounds on Biofilm Development

Further, gravimetrical determinations as well as iron quantification (ICP-OES) verified that iron is somehow stored in the biofilm matrix and not rinsed out with the cultivation medium flowing through. Here, most of the iron was incorporated in biofilms of experiments E1 and E3 (c = 2.5 mg/L Fe^2+^). In detail, the dry matter of E1 and E3 biofilms contained 21% (*w*/*w*) and 50% (*w*/*w*) iron, respectively. In comparison, biofilms of experiments E2 and E4—both performed with low iron(II) concentration of c = 0.25 mg/L Fe^2+^—accumulated 2% (*w*/*w*) and 7% (*w*/*w*) iron in the determined dry matter, respectively.

Several hypotheses were postulated that describe the uptake and influence of iron into the biofilm. For instance, Kang and Kirienko [38] confirmed an uptake of iron via siderophores (iron carriers) as well as the storage of iron in *Pseudomonas aeruginosa* biofilms. Rizzi et al. [64] reported that *Bacillus subtilis* utilizes the formation of biofilms and the production of siderophores to take up iron (Fe) from the medium, likewise, to ensure normal growth. Thereby the authors defined iron (Fe) as the most important metal in biology [64]. Additionally, Oh, Andrews, and Jeon (2018) found out that iron promotes biofilm formation through oxidative stress and that it stimulates EPS production in *Campylobacter jejuni*. Hence, in their study, an addition of iron markedly supported the formation of microcolonies in the early stage as well as the differentiation into mature biofilm structures, which is reflected here both by the OCT as well as the analysis of the structural biofilm parameters.

Additionally, measurements of the individual biofilms via attenuated total reflectance infrared spectroscopy (ATR-IR) were performed (data presented in SI Figure S2). It is visible from SI Figure S2 that experiments E1 and E3 (c = 2.5 mg/L Fe^2+^) as well as E2 and E4 (c = 0.25 mg/L Fe^2+^) are comparable, respectively. These findings are similar to the results of the growth experiment (Figure 2). According to the literature, iron is mainly incorporated into the biofilms via polymorphs of iron oxide-hydroxides (x-FeOOH) [73,74,75,76]. Thereby, in experiments E1 and E3, an incorporation of α-FeOOH took place, whereas in E2 and E4 β-FeOOH was stored into the biofilms. This was found in Wagner [52] and Ivleva et al. [77], too, whereby the authors documented an incorporation of γ-FeOOH into wastewater biofilms. As maintained by Wagner [52] and Möhle et al. [42], the cross-linking of iron with the biofilm matrix ensured an increased stability of the biofilms. α-FeOOH is a highly reactive compound and Omoike et al. [76] reported that an interaction of *Bacillus subtilis* biofilms with α-FeOOH ensures an energetically stable connection for further EPS- and cell adhesion. It was assumed that such an effect made it possible for biofilms in E1 and E3 (c = 2.5 mg/L Fe^2+^) to grow increasingly and to remain stable even at a high flow velocity (u = 3.75 cm/s) without sloughing events of microcolonies. With its loose structure, β-FeOOH exhibited the ability to store high amounts of water [78]. Thus, the low addition of iron(II) in E2 and E4 (c = 0.25 mg/L Fe^2+^) as well as the storage of β-FeOOH into the matrix could be reasons for the reduced accumulation of biofilm mass. Potentially, the type of x-FeOOH incorporation is concentration-dependent and dependent of the bacterial organism or its (cell) surface because the absorptions were clearly distant from each other, as pointed out by Mei et al. [78], too.

It has to be noticed that the study presented here was not intended to reveal the mechanism of iron incorporation into the biofilms cultivated. It thus remains speculative how and why iron was accumulated. However, ATR analysis confirmed the oxidation of Fe^2+^ (inside the cultivation medium) to Fe^3+^ being evenly distributed as α-FeO(OH) within the biofilm matrix. It is supposed that x-FeO(OH) particles/precipitates act like “bridges” for EPS leading to the stabilization of the biofilm matrix and subsequently allowing for the development of thick biofilms even at high wall shear stress levels of 0.27 Pa.

## 4. Conclusions

*Bacillus subtilis* biofilms were cultivated at Fe^2+^ inflow concentrations of 0.25 and 2.5 mg/L, respectively, as well as at wall shear stress levels of 0.05 (low) and 0.27 Pa (high), respectively. It has to be emphasized that this study aimed at understanding biofilm structure development and correlations between Fe^2+^ inflow concentrations and wall shear stress on the mesoscopic biofilm structure rather than revealing a mechanistic understanding of iron(II, III) incorporation into and accumulation within the biofilm matrix.

Structure development was successfully monitored by means of optical coherence tomography and quantified by a large set of ten structural parameters. Three hypotheses were defined in order to evaluate the effect of iron(II) inside the cultivation medium, wall shear stress present during cultivation as well as both parameters on biofilm structure development. Based on the statistical analysis and ten independent experimental runs (*n* = 10), the following conclusions were drawn:

- Iron is—independently of its inflow concentration—incorporated and accumulated within the biofilm matrix as modifications of FeO(OH). In more detail, *Bacillus subtilis* biofilms cultivated in this study showed the accumulation of α- and β-FeO(OH). Where and how Fe^2+^ added to the cultivation medium was oxidized to Fe^3+^ in FeO(OH) accumulated within the biofilm matrix and was not identified;
- Results hinting at the following features: FeO(OH) provides (i) cross-linking abilities and (ii) promotes EPS production, which subsequently may enhance the adhesive as well as cohesive strength of investigated *Bacillus subtilis* model biofilms;
- A positive correlation between the Fe^2+^ inflow concentration and biofilm development/accumulation (acceptance of H_2_) partially compensates for the increased detachment of biofilm at elevated wall shear stress levels. This result has implications on the treatment of biofilms in settings with elevated iron availability;
- Within the range of tested experimental conditions, no effect of the wall shear stress (calculated for the empty channel) on the biofilm structure was statistically approved (rejection of H_1_ and H_3_).

## Figures and Tables

**Table 1:** Overview of the conducted experiments.

Experiment	$c_{Fe}$^2+^ (mg/L)	Q	u (cm/s)	$\tau_{w}$ (Pa)
E1	2.5	1.0	0.75	0.05
E2	0.25	1.0	0.75	0.05
E3	2.5	5.0	3.75	0.27
E4	0.25	5.0	3.75	0.27

**Table 2:** Overview of all structural parameters used as well as their abbreviation, unit and interpretation. Abbreviations: substratum coverage (SC), mean biofilm thickness (${\bar{L}}_{F}$), textural entropy (TE), kurtosis $\left(R_{KU}\right)$, skewness ($R_{SK}$), fractal dimension (FD), angular second moment (ASM), inverse difference moment (IDM) as well as average horizontal (AHRL) and average vertical run lengths (AVRL).

Parameter	Abbr.	Unit	Interpretation
Mean biofilm thickness	${\bar{L}}_{F}$	µm	Biofilm height in z-direction; calculated from the bulk–biofilm interface to the substratum with neglection of pores
Substratum coverage	SC	%	Coverage of the flow cell bottom with biofilm; 100 % minus this parameter would result in global porosity
Textural entropy	TE	-	Is a measure of the randomness of the pixel intensity distribution and thus of the biofilm heterogeneity (increasing values explain more heterogeneous biofilms)
Fractal dimension	FD	-	Describes the irregularity of the aggregates‘ surfaces; higher values equal a higher surface roughness
Skewness	$R_{SK}$	-	Determines the occurrence of low (valleys; $R_{SK}$ < 0) and high biofilm colonies (hills; $R_{SK}$ > 0) in the biofilm structure
Kurtosis	$R_{KU}$	-	Defines the distribution of these occurred valleys and hills on the biofilms’ surface
Average second moment	ASM	-	Direction-orientated indicator of the cell clusters (higher values describe dimensional uniformity; lower values define a change in growth direction (x, y) of the biofilm aggregates)
Inverse difference moment	IDM	-	Similar to ASM but distance-orientated (lower values indicate that distances between biofilm aggregates decrease)
Average vertical run length	AVRL	µm	Mean colony width in y-direction; calculated from separated biofilm aggregates in the flow channel
Average horizontal run length	AHRL	µm	Mean colony length in x-direction; calculated from separated biofilm aggregates in the flow channel

**Table 3:** Two-factorial variance analysis of the calculated structural biofilm parameters shown as p-values. p<α corresponds to a rejection of the hypothesis. H_1_: no difference in structural biofilm parameters due to flow velocities. H_2_: no differences caused by varied Fe^2+^ concentrations. H_3_: no correlation between the two parameters c and u. It has to be noticed that hypotheses were tested for the biofilm structure acquired and analyzed at the end of the experiment (day 10).

Two-Factorial Variance Analysis (*α* = 0.01)			
	***ANOVA***		
**Structural Parameter**	**H_1_**	**H_2_**	**H_3_**
${\bar{L}}_{F}$	0.45	0.90·10^−4^	0.24
SC	0.84	0.10·10^−4^	0.57
TE	0.35	0.30·10^−5^	0.24
$R_{SK}$	0.07	0.18·10^−2^	0.61
FD	0.11	0.12·10^−4^	0.43
ASM	0.63	0.27·10^−5^	0.32
IDM	0.79	0.97·10^−5^	0.55
	***Scheirer-Ray-Hare***		
**Structural parameter**	**H_1_**	**H_2_**	**H_3_**
$R_{KU}$	0.16	0.56·10^−5^	0.21
AHRL	0.17	0.12·10^−6^	0.04
AVRL	0.78	0.17·10^−4^	0.21

## Data Availability

Not applicable.

## Supplementary Materials

The following are available online at https://www.mdpi.com/article/10.3390/microorganisms10112234/s1, Figure S1: Maximum intensity projections (MIPs) of all biofilm replicates of experiment, Figure S2: ATR-IR spectroscopic acquisition of the biofilms on day 10 (incl. band assignment and references).

## Nomenclature

α	significance level
AFM	Atomic Force Microscopy
ANOVA	ANalysis Of VAriance
ATR-IR	Attenuated Total Reflectance Infrared Spectroscopy
c	mass concentration (mg/L)
CLSM	Confocal Laser Scanning Microscopy
E1–E4	Experiments E1 to E4
EDX	Energy-Dispersive X-ray microanalysis
EPS	Extracellular Polymeric Substances
FC	Flow Cell
Fe^2+^	Iron(II)
H_1_–H_3_	Hypotheses 1 to 3 for the ANOVA
ICP-OES	Inductively Coupled Plasma Optical Emission Spectroscopy
LB	Lysogeny Broth (LB-Miller, 10 g/L NaCl)
MIP	Maximum Intensity Projection (height map)
OCT	Optical Coherence Tomography
p	exceedance probability (significance value)
PVC	Poly-Vinyl-Chloride
ROS	Reactive Oxygen Species
Q	volumetric flow rate (mL/min)
SEM	Scanning Electron Microscopy
$\tau_{w}$	wall shear stress in flow cells (Pa)
u	flow velocity (cm/s)
x-FeO(OH)	iron oxide-hydroxide polymorph
