# Supplementary material for: Impact of Fe2+ and Shear Stress on the Development and Mesoscopic Structure of Biofilms—A Bacillus subtilis Case Study
Source: Microorganisms. 2022 Nov 11;10(11):2234. doi: 10.3390/microorganisms10112234 (PMC9699539; doi:10.3390/microorganisms10112234)
Supplement: Supplementary file 1 [file microorganisms-10-02234-s001.zip › Figure S1.pdf]

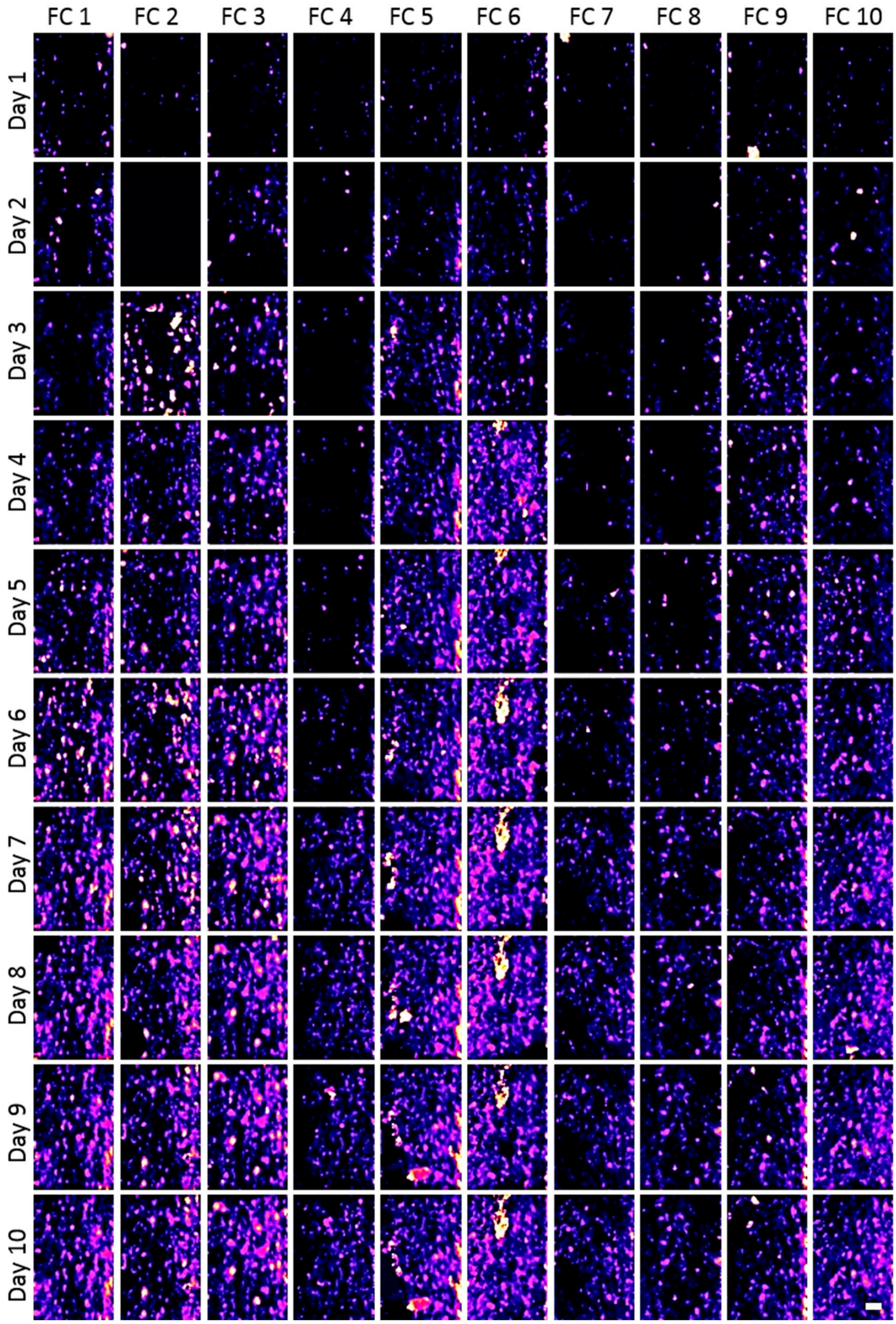

Figure S1 (A): Maximum intensity projections (MIPs) of all biofilm replicates of experiment E1 (c_{Fe2+} = 2.5 mg/L; u = 0.75 cm/s) over the course of the experiment (10 days). The scale bar equals 1 mm. Flow was from top to down.

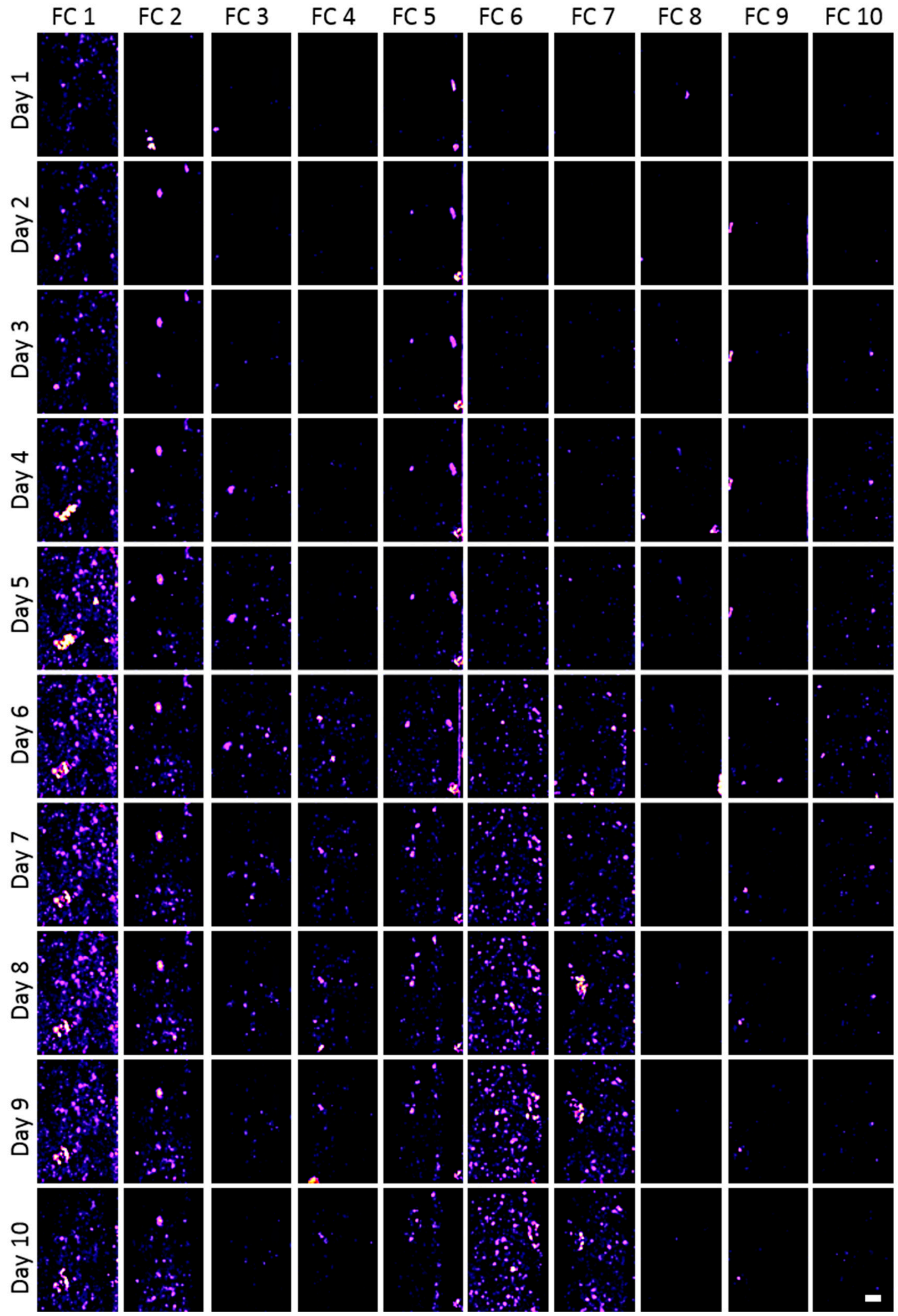

Figure S1 (B): Maximum intensity projections (MIPs) of all biofilm replicates of experiment E2 (c_{Fe2+} = 0.25 mg/L; u = 0.75 cm/s) over the course of the experiment (10 days). The scale bar equals 1 mm. Flow was from top to down.

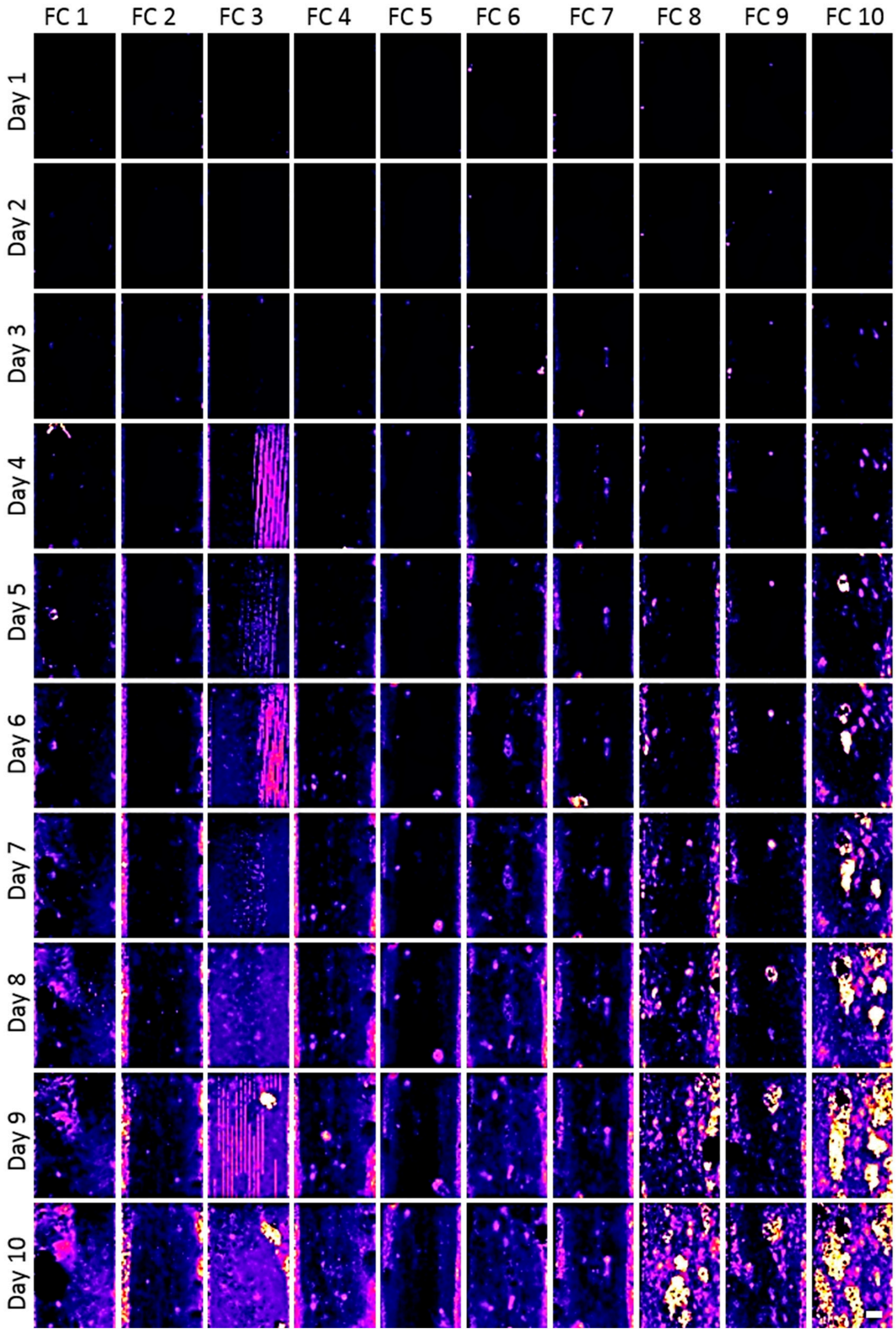

Figure S1 (C): Maximum intensity projections (MIPs) of all biofilm replicates of experiment E3 (c_{Fe2+} = 2.5 mg/L; u = 3.75 cm/s) over the course of the experiment (10 days). The scale bar equals 1 mm. Flow was from top to down.

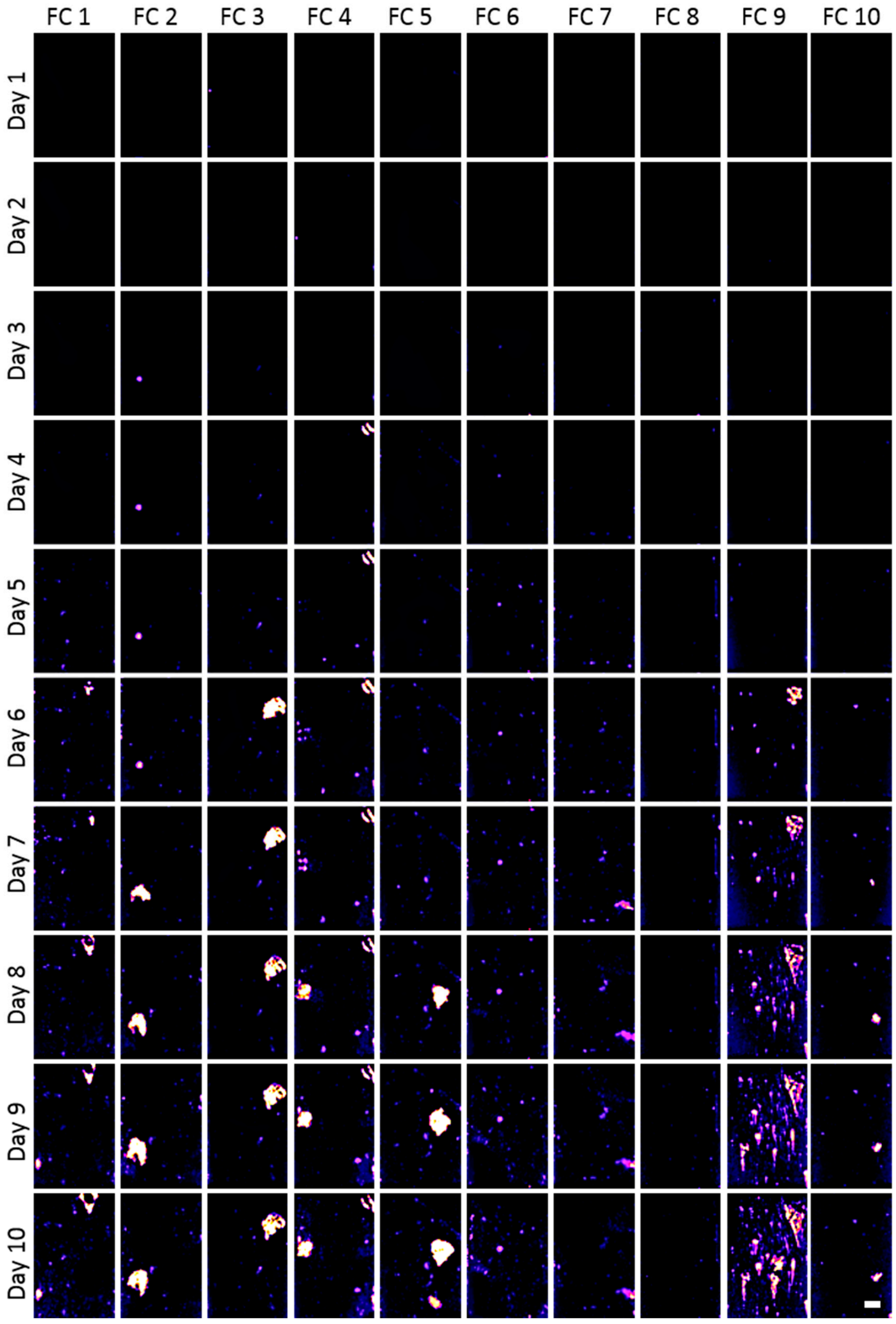

Figure S1 (D): Maximum intensity projections (MIPs) of all biofilm replicates of experiment E4 (c_{Fe2+} = 0.25 mg/L; u = 3.75 cm/s) over the course of the experiment (10 days). The scale bar equals 1 mm. Flow was from top to down.
