# Supplementary material for: Impact of Fe2+ and Shear Stress on the Development and Mesoscopic Structure of Biofilms—A Bacillus subtilis Case Study
Source: Microorganisms. 2022 Nov 11;10(11):2234. doi: 10.3390/microorganisms10112234 (PMC9699539; doi:10.3390/microorganisms10112234)
Supplement: Supplementary file 1 [file microorganisms-10-02234-s001.zip › Figure S2.pdf]

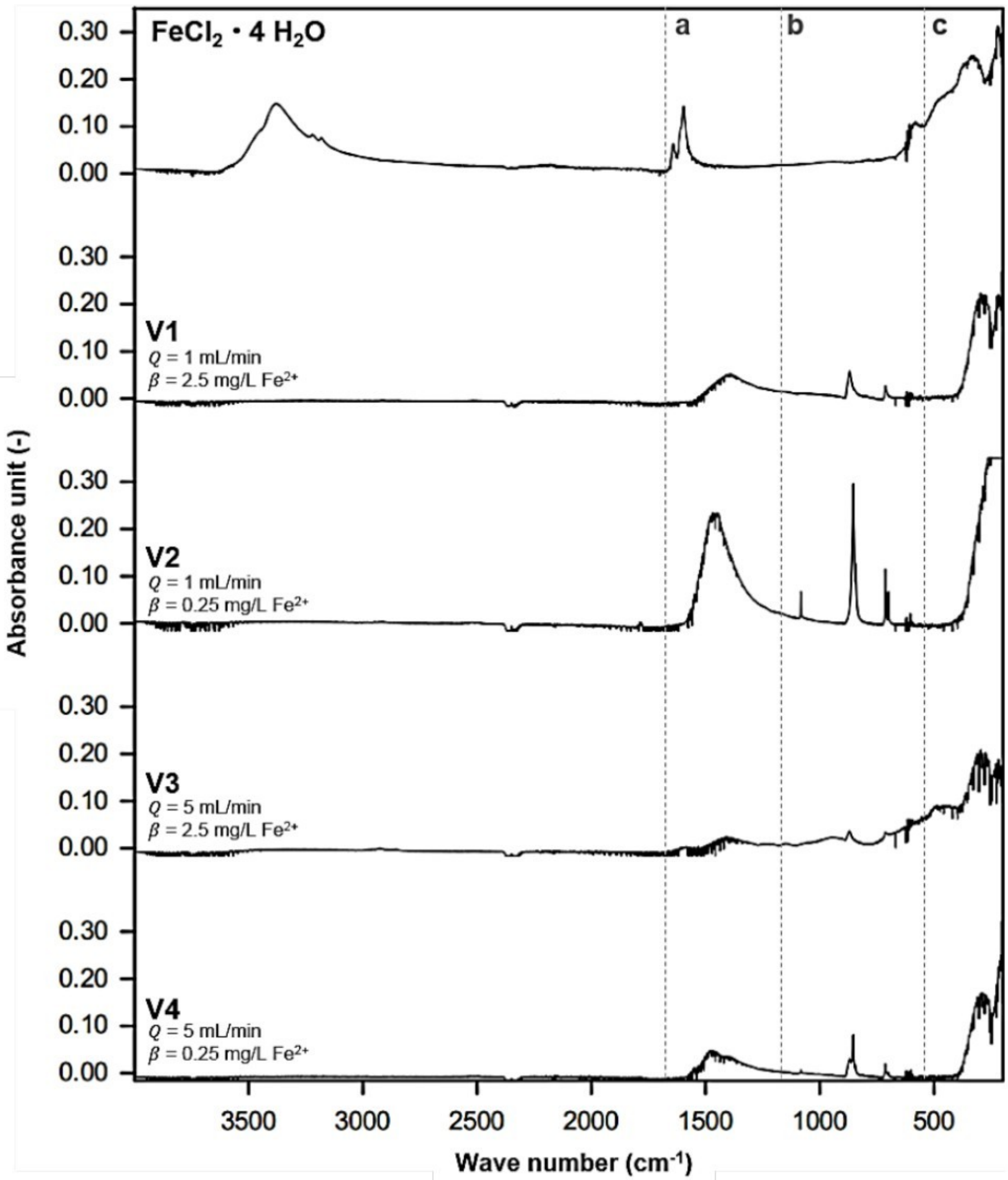

Figure S2: ATR-IR spectroscopic acquisition of the biofilms on day 10. Sections a) - c) indicate characteristic ranges of wave numbers with nearly similar position of the absorptions between the experiments E1 - E4 (1500 - 250 cm^{-1}). $FeCl_2 \cdot 4\ H_20$ served as reference chemical. Annotation: V1 = E1, V2 = E2, V3 = E3, V4 = E4. Band allocation and corresponding references are given below.

**band location experiment (cm^{-1})**	**allocation**	**origin**	**band location literature (cm^{-1})**	**reference**
3378	OH-stretch	Fe-OH	3378	Wei *et al.* (2012)
			3369	Wan Nor *et al.* (2018)
1641	OH-stretch	Fe-OH	1660	Mei *et al.* (2015)
		FeOOH	1630	Dutcher *et al.* (2011)
1595	OH-shift	Romanit	1595	Litescu *et al.* (2012)
1585	COO-Fe	Fe_3O_4 nanoparticle	1587	Wei *et al.* (2011)
1479	CH_2		1479	Unger (2009)
1468	CH_2		1466	Litescu *et al.* (2012)
1416	COO-Fe	Fe_3O_4 nanoparticle	1393	Wei *et al.* (2012)
1392	-CH=CH-	Fe_3O_4 nanoparticle	1412	Wei *et al.* (2012)
			1384	
1083	P-O-stretch	Iron oxide	900-1200	Benyettou *et al.* (2012)
	C-O-C-stretch		1080	Filip *et al.* (2004)
	Fe-OH-bend		1076	
	C-O	Eisen(III)phthalocyanin	1050-1100	Luther & Günzler (1955)
871	OH-bend		865-894	Namduri & Nasrazadani (2008)
870	OH···O	α-FeO(OH)	883	Mei *et al.* (2015)
870			960-870	Salzer *et al.*
	Fe-O-Fe	Fe_2O_3	863	Ulfa & Ulfa (2019)
853	OH-bend	β-FeO(OH)	847	Mei *et al.* (2015)
853	-H-		850	Luther & Günzler (1955)
	C-N		830-920	
716	C=O		750-620	Litescu *et al.* (2012)
714	C-C=O			
713				
700	C-H		900-700	Luther & Günzler (1955)
	NO_2		680-740	
	OH-bend	-FeOOH	696	Mei *et al.* (2015)
582	Fe-O	Fe_3O_4-Nanopartikel	583	Sodipo & Azlan (2015)
			580	Wei *et al.* (2011)
			577	Hwang *et al.* (2014)
493	Fe-O-stretch	α-FeO(OH)	499	Malathi *et al.* (2017)
477	Fe-F	iron(III) phthalocyanine	475	Kalz & Homborg (1983)
297	Fe-Cl	iron(III) phthalocyanine	303	Kalz & Homborg (1983)
286				
236	Fe-Br	iron(III) phthalocyanine	221	Kalz & Homborg (1983)
217				
203	Fe-I	iron(III) phthalocyanine	193	Kalz & Homborg (1983)

References used in Figure S2 in SI

Yan Wei, Bing Han, Xiaoyang Hu, Yuanhua Lin, Xinzhi Wang, Xuliang Deng, Synthesis of Fe3O4 Nanoparticles and their Magnetic Properties, Procedia Engineering, Volume 27, 2012, Pages 632-637, ISSN 1877-7058, https://doi.org/10.1016/j.proeng.2011.12.498.

Wan Nor, W. F. K., Soh, S. K. C., Azmi, A. A. A. R., Yusof, M. S. M. & Shamsuddin, M. Synthesis and physicochemical properties of magnetite nanoparticles (Fe3O4) as potential solid support for homogeneous catalysts. Malaysian J. Anal. Sci. 22, 768–774 (2018).

Mei, L., Liao, L., Wang, Z. & Xu, C. Interactions between phosphoric/tannic acid and different forms of FeOOH. Adv. Mater. Sci. Eng. 2015, 1–10 (2015).

Dutcher, B. u. a. Use of nanoporous FeOOH as a catalytic support for NaHCO3 decomposition aimed at reduction of energy requirement of Na2CO 3/NaHCO3 based CO2 separation technology. J. Phys. Chem. C 115, 15532–15544 (2011).

Litescu, S.-C., Teodor, E. D., Truica, G.-I., Tache, A. & Radu, G.-L. Fourier Transform Infrared Spectroscopy – Useful Analytical Tool for Non-Destructive Analysis. in Infrared Spectroscopy - Materials Science, Engineering and Technology 510 (2012). doi:10.5772/36428

Unger, M. Infrarot-spektroskopische Untersuchungen zur Charakterisierung der thermischen und mechanischen Eigenschaften biologisch abbaubarer Polymere. (Duisburg-Essen, 2009).

Benyettou, F., Milosevic, I., Motte, L. & Olsen, J. Ultra-Small Superparamagnetic Iron Oxide Nanoparticles Made to Order. J. Bioanal. Biomed. S5, 1–6 (2012).

Filip, Z., Hermann, S. & Demnerová, K. FT-IR spectroscopic characteristics of differently cultivated Escherichia coli. Czech J. Food Sci. 26, 458–463 (2008).

Luther, H. & Günzler, H. Die Molekülschwingungsspektren des Naphthalins und seiner Derivate. Zeitschrift für Naturforsch. - Sect. B J. Chem. Sci. 445–457 (1955). doi:10.1515/znb-1955-0808

Namduri, H. & Nasrazadani, S. Quantitative analysis of iron oxides using Fourier transform infrared spectrophotometry. Corros. Sci. 50, 2493–2497 (2008).

Salzer, R., Thiele, S., Suemmchen, L. & Machill, S. ; online: http://www.chemgapedia.de/vsengine/vlu/vsc/de/ch/3/anc/ir_spek/cho_verbindungen.vlu/Page/vsc/de/ch/3/anc/ir_spek/schwspek/spektreninterpretation/ir_13_4_7_3/carbonacid.vscml/Supplement/5.html, accessed on 28.11.2021

Ulfa, D. K. & Ulfa, M. Kinetics Performance for Cadmium (II) Ion Adsorption using Mesoporous Silica Embedded with Iron Oxide. Orient. J. Chem. 35, 20–27 (2019).

Sodipo, B. K. & Azlan, A. A. Superparamagnetic iron oxide nanoparticles incorporated into silica nanoparticles by inelastic collision via ultrasonic field: Role of colloidal stability. AIP Conf. Proc. 1657, (2015).

Hwang, S. W., Umar, A., Dar, G. N., Kim, S. H. & Badran, R. I. Synthesis and characterization of iron oxide nanoparticles for phenyl hydrazine sensor applications. Sens. Lett. 12, 97–101 (2014).

Malathi, A., Arunachalam, P., Madhavan, J., Al-Mayouf, A. M. & Ghanem, M. A. Rod-on-flake α-FeOOH/BiOI nanocomposite: Facile synthesis, characterization and enhanced photocatalytic performance. Colloids Surfaces A Physicochem. Eng. Asp. 435–445 (2018). doi:10.1016/j.colsurfa.2017.10.036

Kalz, W. & Homborg, H. Eisen(III)phthalocyanine: Darstellung und spektroskopische Charakterisierung von [Fe(OR)2Pc(—2)]~ (R = H, CH3, C2H5, (CH3)2CH, (CH3)3C), (FePc(—2))20 und FeXPc(—2) (X = F, Cl, Br, I). Zeitschrift für Naturforsch. 38b, 470–484 (1983).
